# Islet autoantibodies in Thai individuals diagnosed with type 1 diabetes before 30 years of age: a large multicentre nationwide study

**DOI:** 10.1007/s00125-025-06373-y

**Published:** 2025-02-19

**Authors:** Nattachet Plengvidhya, Sarocha Suthon, Tassanee Nakdontri, Nipaporn Teerawattanapong, Saranya Ingnang, Watip Tangjittipokin, Thai Type 1 Diabetes and Diabetes Diagnosed Before Age 30 Years Registry, Care, and Network (T1DDAR CN)

**Affiliations:** 1 Division of Endocrinology and Metabolism, Faculty of Medicine Siriraj Hospital, Mahidol University, Bangkok, Thailand; 2 Siriraj Center of Research Excellence for Diabetes and Obesity, Faculty of Medicine Siriraj Hospital, Mahidol University, Bangkok, Thailand; 3 Siriraj Center of Research Excellence Management, Faculty of Medicine Siriraj Hospital, Mahidol University, Bangkok, Thailand; 4 Research Division, Faculty of Medicine Siriraj Hospital, Mahidol University, Bangkok, Thailand; 5 Department of Immunology, Faculty of Medicine Siriraj Hospital, Mahidol University, Bangkok, Thailand

**Keywords:** Idiopathic type 1 diabetes, Islet autoantibody, Long-standing type 1 diabetes, Thailand, Type 1 diabetes

## Abstract

**Aims/hypothesis:**

Type 1 diabetes is categorised into autoantibody positive and autoantibody negative. Most type 1 diabetes research has focused on European populations, leaving a gap in understanding in relation to other ethnic groups, including Thai populations. This lack of data is significant given Thailand’s poor prevention and therapeutic management strategies. We aimed to investigate the frequency and distribution of islet autoantibodies among Thai individuals with long-standing type 1 diabetes diagnosed before the age of 30 years.

**Methods:**

We conducted a nationwide population-based study involving 48 hospitals in Thailand from May 2020 to September 2023, enrolling 953 participants. Demographic and clinical characteristics of individuals with autoantibody-positive and -negative type 1 diabetes were analysed. The autoantibodies GAD65, IA-2 and ZnT8 were measured using ELISA. A random C-peptide level was detected by electrochemiluminescence immunoassay.

**Results:**

Thai individuals with autoantibody-negative type 1 diabetes comprised 34.2% of the population. Among all individuals, the frequency of GAD65, IA-2 and ZnT8 was 56%, 37% and 33%, respectively. Autoantibody-negative individuals with type 1 diabetes were older at diagnosis, had higher BMI and had higher random C-peptide levels compared with autoantibody-positive individuals with type 1 diabetes. Female individuals had a higher prevalence of type 1 diabetes than male individuals (58% vs 42%; *p*=1.531 × 10^−5^). The southern region of Thailand exhibited a distinct pattern of autoantibody frequency compared with other regions (*p*=0.0001561).

**Conclusions/interpretation:**

The frequency, distribution and characteristics of autoantibody-positive and -negative long-standing type 1 diabetes in Thailand showed uniqueness from other populations. This provides insight into the disease that may have implications for type 1 diabetes prediction, treatment and pathogenesis, especially in the Southeast Asian population.

**Graphical Abstract:**

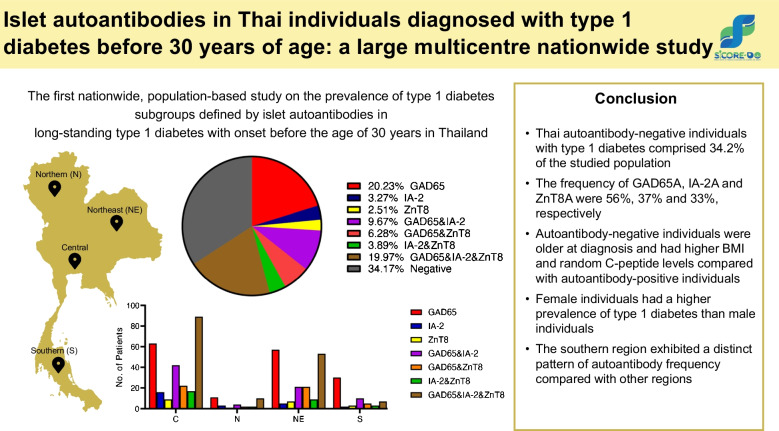

**Supplementary Information:**

The online version of this article (10.1007/s00125-025-06373-y) contains peer-reviewed but unedited supplementary material.


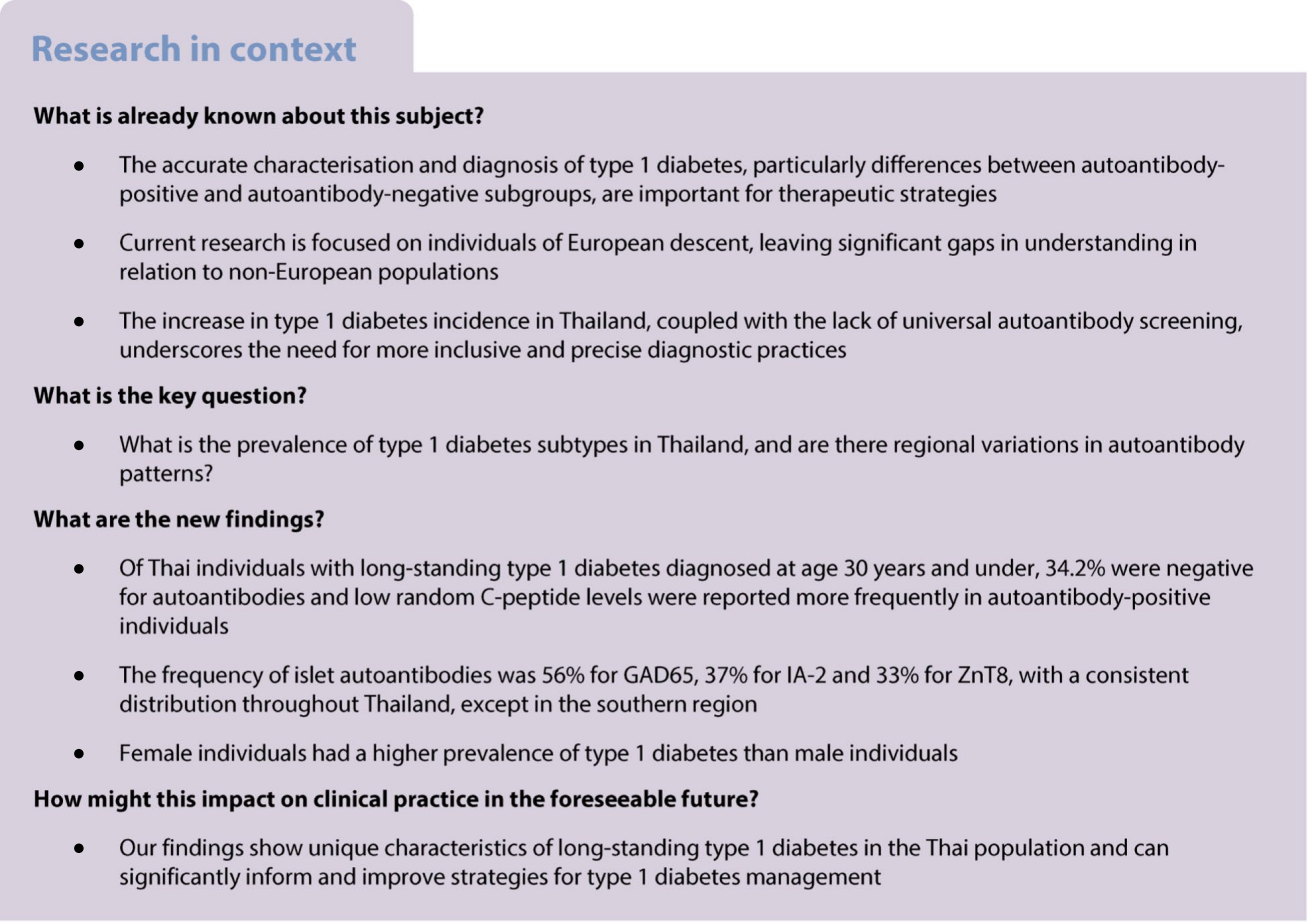


## Introduction

Type 1 diabetes is characterised by autoimmune destruction of pancreatic beta cells, resulting in complete insulin deficiency. This disease is subdivided into two subgroups based on the presence of autoantibodies: autoantibody-positive and autoantibody-negative type 1 diabetes [1]. Most individuals with type 1 diabetes belong to the autoantibody-positive group, marked by the presence of autoantibodies against islet antigens such as GAD65, tyrosine phosphatase (IA-2) and ZnT8. On the contrary, approximately 10–15% of individuals with type 1 diabetes are classified as having autoantibody-negative type 1 diabetes, diagnosed without detectable autoantibodies. There are distinct differences in the metabolic, clinical and immune profiles between these two subgroups [2]. Autoantibody-positive type 1 diabetes is often found in individuals of non-European ethnicity; however, autoantibody-negative type 1 diabetes is associated with older age at diagnosis, non-European ethnicity and lack of associations with human leukocyte antigen [3]. Fifty-eight per cent of individuals with type 1 diabetes are diagnosed at the age of 30 years or younger [4], increasing the risk of long-term complications such as diabetic ketoacidosis (DKA), retinopathy, nephropathy, neuropathy and CVD [5, 6].

Accurate characterisation of individuals with type 1 diabetes, particularly the detection and analysis of pancreatic autoantibodies, is crucial for precise diagnosis and treatment selection. Existing research focuses predominantly on individuals of European descent, leaving gaps in understanding in relation to non-European populations [7]. Inadequate diagnosis and treatment has led Southeast Asia to have a high type 1 diabetes mortality rate at young age [8]. In Thailand, the annual incidence rate of type 1 diabetes increased by 12.5% from 2015 to 2020 [9]; however, antibody screening in individuals with type 1 diabetes is not yet covered by a Universal Coverage Policy. Moreover, comprehensive data on the frequency and distribution of islet autoantibodies across diverse geographic regions are lacking, leading to the exclusion of some individuals from accurate diagnosis and adequate treatment. Thus, a precise diagnosis can significantly impact the management and treatment of type 1 diabetes, helping affected individuals to maintain a better quality of life, and is crucial to advancing the Sustainable Development Goals (SDGs) of the United Nations and building human capital [10].

Our study aims to investigate the prevalence of islet autoantibodies in long-standing type 1 diabetes among Thai individuals diagnosed at 30 years of age or younger. By being the first to examine a diverse sample from various Thai regions, we aim to provide comprehensive information on autoimmune profiles of type 1 diabetes in Thailand. Addressing these knowledge gaps will inform future diagnostic and therapeutic strategies, especially in the Asian population.

## Methods

### Study design and participants

We planned to recruit 1000 individuals diagnosed with type 1 diabetes in Thailand from node hospitals together with the Thai Type 1 Diabetes and Diabetes Diagnosed Before Age 30 Years Registry, Care, and Network (T1DDAR CN). These hospitals function as referral centres for local hospitals located within their referral area, mainly divided into four regions as follows: central, northern, northeast and southern. Each region represents the type and characteristics of individuals with type 1 diabetes according to their home address.

Type 1 diabetes was diagnosed according to ADA criteria [11]. Autoantibody-positive type 1 diabetes was diagnosed based on the presence of diabetes in conjunction with one or more of the following criteria: the detection of autoantibodies against islet cell antigens, such as GAD, IA-2, ZnT8 or a combination of these antibodies; a random C-peptide level of <0.20 nmol/l or a diagnosis of DKA. DKA was defined by meeting the following conditions: a blood glucose concentration >11.0 mmol/l or a known history of diabetes mellitus; a capillary or blood ketone concentration >3.0 mmol/l or significant ketonuria (measured as 2+ or more on standard urine dipsticks); a bicarbonate concentration <15 mmol/l or a venous pH of less than 7.3; and an anion gap calculated as (Na^+^ + K^+^) − (Cl^−^ + HCO_3_^−^) concentration >10 [12]. The diagnosis of autoantibody-negative type 1 diabetes required meeting the following criteria [13]: diagnosis of type 1 diabetes; negativity for three autoantibodies; and exclusion of monogenic diabetes. Individuals who had clinical characteristics consistent with type 2 diabetes or MODY were excluded from this study.

**Fig. 1:**
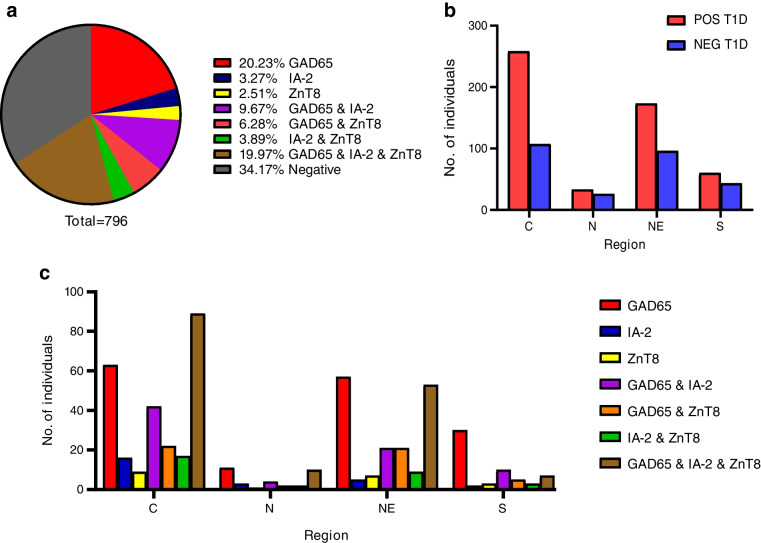
Percentage and distribution of autoantibodies in Thai individuals diagnosed with type 1 diabetes before the age of 30 years. (**a**) Percentage of autoantibodies detected in the total study population of Thai individuals with type 1 diabetes. (**b**) Distribution of autoantibody-positive and autoantibody-negative individuals by region. (**c**) Frequency of autoantibodies according to region. C, central; N, northern; NE, northeast; S, southern; NEG T1D, autoantibody-negative individuals with type 1 diabetes; POS T1D, autoantibody-positive individuals with type 1 diabetes

This study was a cross-sectional investigation conducted from May 2020 to September 2023. It included individuals diagnosed with type 1 diabetes across all age groups, as well as individuals who were diagnosed with diabetes before the age of 30 years. A total of 953 individuals with confirmed type 1 diabetes were recruited for participation. The process and criteria for the selection of these individuals are detailed in electronic supplementary material (ESM) Fig. 1.

The entire study was conducted according to the Ethics Guidelines of the Declaration of Helsinki and was approved by the Siriraj Institutional Review Board, Siriraj Hospital Faculty of Medicine, Mahidol University, Bangkok, Thailand (certificate of approval no. SI 491/2014).

### Clinical characteristics and biochemical measurements

The staff at each participating hospital received training to standardise data collection. Demographic data, including sex, age at diagnosis, body weight and height, were collected at baseline. Sex and gender were considered in the study design following SAGER (Sex and Gender Equity in Research) guidelines. Sex of participants was assigned at birth. Peripheral blood was collected by trained nurses and sent to Siriraj Hospital Faculty of Medicine, Mahidol University, Bangkok within 24 h. Serum or plasma samples were isolated and stored at −80℃ until biochemical measurements were performed. GAD65, IA-2 and ZnT8 autoantibodies were detected by ELISA (RSR, Cardiff, UK) at the Laboratory of the Division of Endocrinology and Metabolism, Department of Paediatrics, Faculty of Medicine, Siriraj Hospital, Mahidol University. The sensitivity and specificity for GAD65, IA-2 and ZnT8 assays were accorded to the Diabetes Antibody Standardisation Program [14, 15]. The manufacturer’s cut-off values for determining positivity had been established as follows: ≥5 U/ml for GAD65; ≥7.5 U/ml for IA-2; and ≥15 U/ml for ZnT8. Plasma C-peptide levels were measured using an electrochemiluminescence immunoassay (Roche Diagnostic, Mannheim, Germany) at the Laboratory of the Department of Clinical Pathology, Faculty of Medicine, Siriraj Hospital, Mahidol University. A low random C-peptide level was considered at <0.20 nmol/l [16, 17].

### Statistical analysis

All analyses were performed using R version 4.4.0 (https://cran.rstudio.com/). The categorical variables are presented as proportions or percentages and compared using the χ^2^ test. Continuous variables are presented as mean ± SD or as median (IQR [Q1–Q3]). The distribution of the variables was assessed for normality by the Shapiro–Wilk test before comparison. The Mann–Whitney *U* test was employed for comparisons between two independent groups. Kernel density estimation (KDE) was used to evaluate the unimodal distribution pattern. Statistical significance was considered as *p*≤0.05.

## Results

### Baseline characteristics of Thai individuals with type 1 diabetes

The demographic and clinical characteristics of the individuals with type 1 diabetes are shown in Table 1. It was observed that autoantibody-negative individuals had a significantly longer duration of disease than their autoantibody-positive counterparts. Specifically, the 75th percentile of the duration within which autoantibodies were detected among autoantibody-positive individuals was 6.91 years. In contrast, this duration extended to 9.08 years for autoantibody-negative individuals, indicating that the probability of failing to detect all antibodies was low in these individuals.

**Table 1:** Characteristics of autoantibody-positive and autoantibody-negative individuals with type 1 diabetes

Characteristic	Autoantibody positive	Autoantibody negative	*p* value
No. of individuals	524 (65.8)	272 (34.2)	0.0016^a^
Age at diagnosis, years	11.6 ± 6.7	15.4 ± 6.6	3.246 × 10^−16b^
Duration of diabetes, years	1.63 (0.23–6.91)	4.14 (0.87–9.08)	6.081 × 10^−6b^
BMI^c^, kg/m^2^	19.54 ± 4.85	23.41 ± 5.46	<2.2 × 10^−16b^
DKA history^d^	287 (74.3)	120 (61.5)	0.272^a^
Random C-peptide level, nmol/l	0.03 (0.01–0.14)	0.21 (0.03–0.54)	<2.2 × 10^−16b^
Report of a low random C-peptide level	434 (76)	134 (24)	<2.2 × 10^−16a^

Data are mean ± SD, median (IQR) or *n* (%)

^a^χ^2^ square test

^b^Kruskal–Wallis test

^c^The total number of individuals for BMI was 726

^d^DKA history was available for 386 autoantibody-positive individuals and 195 autoantibody-negative individuals

The prevalence of autoantibody-positive type 1 diabetes was significantly higher than that of autoantibody-negative type 1 diabetes (65.8% vs 34.2%; *p*=0.0016). Autoantibody-positive individuals with type 1 diabetes were diagnosed at a significantly younger age compared with autoantibody-negative individuals with type 1 diabetes (mean ± SD 11.6 ± 6.7 vs 15.4 ± 6.6 years; *p*=3.24 × 10^−16^). When dividing the age into childhood (0–17 years) and young adulthood (18–30 years), the number of individuals in the childhood group was higher than in the young adulthood group when considering type 1 diabetes overall and its autoantibody-positive and -negative subgroups (ESM Table 1). Furthermore, BMI was significantly lower in autoantibody-positive individuals with type 1 diabetes than in autoantibody-negative individuals with type 1 diabetes (mean ± SD 19.54 ± 4.85 vs 23.41 ± 5.46 kg/m^2^; *p*<2.2 × 10^−16^). The record of DKA at any point after type 1 diabetes diagnosis was not significantly different between autoantibody-positive and autoantibody-negative individuals with type 1 diabetes.

### Autoantibody frequency and distribution in Thai individuals

In Thai individuals diagnosed with diabetes before the age of 30 years, the frequency of each autoantibody was 56%, 37% and 33% for GAD65, IA-2 and ZnT8, respectively (Table 2). When autoantibody status was categorised into negative, single and combinations, the number of autoantibodies was significantly different between each category (Fig. 1a; *p*<2.2 × 10^−16^). The KDE plots of GAD65, IA-2 and ZnT8 showed a unimodal pattern in the Thai population (ESM Fig. 2a–c) and there was no significant difference in autoantibody levels between sexes (ESM Fig. 2d–f).

**Table 2:** Specific autoantibody frequency

Specific autoantibody	Number (% of all individuals with type 1 diabetes)
GAD65	447 (56)
IA-2	293 (37)
ZnT8	260 (33)

The number of individuals with autoantibody-positive type 1 diabetes and autoantibody-negative type 1 diabetes varied significantly between Thai regions (Fig. 1b; *p*=0.02557). The percentage of individuals with C-peptide levels <0.20 nmol/l in the type 1 diabetes subgroups differed significantly (Table 1; 76% vs 24%; *p*<2.2 × 10^−16^); however, no statistically significant difference was found when comparing the type 1 diabetes subgroups between regions (ESM Fig. 3; *p*=0.2237).

The number of individuals positive for each autoantibody or combinations of the autoantibodies showed significant differences between regions (Fig. 1c; *p*=0.03943). The northern and northeast regions had a similar autoantibody distribution, with no significant differences between the GAD65+IA-2+ZnT8 frequency and the GAD65 frequency. In particular, the central and southern regions exhibited a different frequency of the autoantibodies within the region (central, *p*=0.03496; southern, *p*=0.0001561); however, the direction of these differences was opposite (ESM Table 2).

### The association between sex and type 1 diabetes subgroups

Thai female individuals were diagnosed with type 1 diabetes significantly more often than male individuals (Table 3; 58% vs 42%; *p*=1.531 × 10^−5^). Similarly, the proportion of female individuals with autoantibody-positive and autoantibody-negative type 1 diabetes was significantly higher than in male individuals (Table 3; respectively, 58.2% vs 41.8% [*p*=0.000172] and 56.6% vs 43.4% [*p*=0.02905]). There was no significant difference in the history of DKA at any point after type 1 diabetes diagnosis when comparing the male sex and the female sex.

**Table 3:** Sex distribution of type 1 diabetes and subgroups

Type 1 diabetes group	Male sex	Female sex	*p* value^a^
Type 1 diabetes, *n* (%)	337 (42)	459 (58)	1.531 × 10^−5^
Autoantibody-positive type 1 diabetes, *n* (%)	219 (41.8)	305 (58.2)	0.000172
Autoantibody-negative type 1 diabetes, *n* (%)	118 (43.4)	154 (56.6)	0.02905
DKA history^b^, *n* (% of individuals in each sex)	238 (70.6)	169 (69.3)	0.9125

^a^χ^2^ test

^b^DKA history was available for 337 male individuals and 244 female individuals

## Discussion

Our analysis presents the first nationwide population-based study on the prevalence of type 1 diabetes subgroups based on islet autoantibodies with diagnosis before the age of 30 years in Thailand. It reveals that 34.2% of the Thai individuals with type 1 diabetes were autoantibody-negative, a considerably higher proportion than found in other Asian populations [18, 19] or in European populations [20–23].

The demographic and clinical characteristics of individuals in this study supported the distinct mechanisms of immune-mediated pancreatic beta cell damage in the type 1 diabetes subgroups. Low random C-peptide levels (less than 0.20 nmol/l) were found predominantly in autoantibody-positive type 1 diabetes compared with autoantibody-negative type 1 diabetes [18, 24]. Autoantibody-negative individuals were older at diagnosis and had higher BMI than autoantibody-positive individuals, consistent with other reports [18, 22]. DKA in diagnosis of type 1 diabetes was less common in autoantibody-negative individuals with type 1 diabetes compared with their counterparts [18, 19, 21, 23]. However, our study did not observe any significant differences in the history of DKA between individuals with autoantibody-negative type 1 diabetes and their autoantibody-positive counterparts, nor were there notable differences when comparing male and female individuals [22]. Notably, the crude DKA incidence rate in Thai individuals with type 1 diabetes was 10.8%, with the rate being higher in the female sex than in the male sex [9].

The distribution of autoantibody-positive and -negative type 1 diabetes was similar in all regions of Thailand. Notably, the southern region exhibited a unique pattern. This could be due to the distinct population diversity in the southern region of Thailand [25, 26]. Furthermore, the frequencies of GAD65 (56%), IA-2 (37%) and ZnT8 (33%) were lower than those reported in Asian and European individuals with type 1 diabetes [23, 27, 28]. This may affect the distributions of all autoantibodies that were unimodal, differing from those in the Chinese and UK populations [29, 30]. Notably, a previous study involving Thai juveniles diagnosed with type 1 diabetes at the age of 15 years or younger showed a higher prevalence of those autoantibodies compared with this study [31].

Type 1 diabetes is generally more common in male than in female individuals in various ethnicities [18, 23, 32, 33]. Interestingly, we found that type 1 diabetes occurred more frequently in the female sex than in the male sex, consistent with previous reports in Thailand [9, 31, 34]. Moreover, there was a sex predominance in both subgroups of type 1 diabetes, with female individuals having a higher frequency than male individuals. In contrast, studies in other populations did not show differences between the sexes in the prevalence of islet antibody-negative individuals [18, 19, 21]. While sex differences can influence the level of autoantibodies [35, 36], no such association was found in Thai individuals with type 1 diabetes. This may be due to the low levels of autoantibodies in the population.

We acknowledge several limitations in our national population-based study. First, the difference in the prevalence of autoantibody-positive and autoantibody-negative type 1 diabetes should be interpreted with caution. Since the enrolled individuals were not newly diagnosed, it is possible that some autoantibody-negative individuals might have tested positive if autoantibodies were measured at the time of diagnosis. Additionally, insulin autoantibody was not assessed, and some of the autoantibody-negative individuals may have had this antibody at diagnosis. However, previous studies have shown that autoantibodies can still be detected in individuals with very long-standing type 1 diabetes [37, 38]. Second, we were unable to collect data on additional biochemical variables (such as HbA_1c_, fasting plasma glucose and creatinine) nor on other complications (such as diabetic retinopathy and nephropathy), which could have differed between autoantibody-positive and autoantibody-negative type 1 diabetes. Future research on the association of type 1 diabetes subgroups with other complications will improve our understanding of disease pathogenesis. Lastly, the individuals’ regional data was classified based on their living address rather than their birthplace, so could not accurately represent their region of origin and the effect of genetics.

Taken together, we reveal the prevalence, distribution and characteristics of long-standing autoantibody-positive and -negative type 1 diabetes in Thailand, representing the first study of type 1 diabetes subgroups in the Southeast Asian population. Locally, our findings provide evidence for healthcare systems to reconsider the criteria for type 1 diabetes autoantibody detection, ensuring that all individuals receive standardised and effective treatment. However, studies in individuals with new-onset disease are needed to confirm our findings. Globally, this enhances the understanding of type 1 diabetes beyond European and East Asian populations, opening the way for future research on the prediction, treatment and prognosis of type 1 diabetes in diverse populations.

## Electronic supplementary material

Below is the link to the electronic supplementary material.

ESM (PDF 743 KB)

## Data Availability

The datasets generated during and/or analysed in the current study are available from the corresponding author upon reasonable request.

## Appendix

The following people participated in the T1DDAR CN:

**Central region**

**University Hospitals:** Siriraj Hospital, Mahidol University, Bangkok (Apiradee Sriwijitkamol, Jeerunda Santiprabhob, Lukana Preechasuk, Ornsuda Lertbannaphong, Raweewan Lertwattanarak, Supawadee Likitmaskul); The Golden Jubilee Medical Center, Nakhonpathom (Ornwimon Mukpaksacharoen) Thammasat University Hospital, Pathum Thani (Thipaporn Tharavanij); Vajira Hospital, Navamindradhiraj University, Bangkok (Natphassorn Dermkhuntod, Petch Rawdaree).

**Hospitals in the Ministry of Public Health:** Charoenkrung Pracharak Hospital, Bangkok (Phatharaporn Kiatpanabhikul, Supawut Suksantilirs); Queen Sirikit National Institute of Child Health, Bangkok (Chawkaew Kongkanka, Piriya Chantrathammachart); Rajavithi Hospital, College of Medicine, Rangsit University, Bangkok (Chaicharn Deerochanawong); Taksin Hospital, Bangkok (Worraporn Tantichattanon). Hospitals in the Ministry of Defense: Bhumibol Adulyadej Hospital, Bangkok (Nattapol Sathavarodom); Takhli Hospital, Nakhon Sawan(Tanuttiya Arunothong); HRH Princess Maha Chakri Sirindhorn Medical Center- MSMC Hospital, Nakhon Nayok (Nattakarn Wongjitrat); Nakhonpathom Hospital, Nakhonpathom (Pimonsri Hantanasiriskul); Nong Chang Hospital, Uthai Thani (Anchana Jongkhetkarn).

**Northern region**

**University Hospitals:** Chiang Mai University Hospital, Chiang Mai (Danil Wongsa, Prapai Dejkhamron).

**Hospitals in the Ministry of Public Health:** Buddhachinaraj Hospital, Phitsanulok (Meijinee Densriwiwat); Chiangrai Prachanukroh Hospital, Chiang Rai City (Napatsorn Sarattana); Nakornping Hospital, Chiang Mai (Hataitip Tangngam, Tattiwa Nirach); Nan Hospital, Nan (Srisattaya Ponlarat); Phayao Hospital, Phayao (Tassimon Pattaropong); Uttaradit Hospital, Uttaradit (Sarut Kontha).

**Northeast region**

**University Hospitals:** Srinagarind Hospital, Khon Kaen University, Khon Kaen (Suranut Charoensri).

**Hospitals in the Ministry of Public Health:** Khon Kaen Hospital, Khon Kaen (Chatchai Suesirisawad, Nattakarn Suwansaksri); Maharat Nakhon Ratchasima Hospital, Nakhon Ratchasima (Priya Sanguanwongwichit, Puntip Tantiwong, Sirilak Setthalak); Sakon Nakhon Hospital (Narissara Sunon, Wasana Sanmahachai); Phonthong Hospital, Roi-Et (Thapanee Ketklieng), Sisaket Hospital, Sisaket (Sathaporn Hin Ngern, Chompunuch Samanchart), Mahasarakham Hospital, Mahasarakham (Yukuntorn Thapimai); Chaiyaphum Hospital, Chaiyaphum (Thanissak Taweekote) Chonburi Hospital (Panuwat Suteerayongprasert); Prapokklao Hospital, Chanthaburi (Thapana Roonghiranwat, Napaporn Keawyu); Rayong Hospital, Rayong (Chotima Sornsiriwong, Tippawan Kongvitayanon).

**Southern region**

**Hospitals in the Ministry of Public Health:** Maharaj Nakhon Si Thammarat Hospital, Nakhon Si Thammarat (Saowanee Nakkaew); Surat Thani Hospital, Surat Thani (Palinee Nantarakchaikul); Vachira Phuket Hospital, Phuket (Attasit Rungrojsakorn).

## Abbreviations

DKA: Diabetic ketoacidosis
KDE: Kernel density estimation
T1DDAR CN: Thai Type 1 Diabetes and Diabetes Diagnosed Before Age 30 Years Registry, Care, and Network
