# Correction: Islet autoantibodies in Thai individuals diagnosed with type 1 diabetes before 30 years of age: a large multicentre nationwide study

**DOI:** 10.1007/s00125-025-06404-8

**Published:** 2025-03-10

**Authors:** Nattachet Plengvidhya, Sarocha Suthon, Tassanee Nakdontri, Nipaporn Teerawattanapong, Saranya Ingnang, Watip Tangjittipokin, Thai Type 1 Diabetes and Diabetes Diagnosed Before Age 30 Years Registry, Care, and Network (T1DDAR CN)

**Affiliations:** 1 Division of Endocrinology and Metabolism, Faculty of Medicine Siriraj Hospital, Mahidol University, Bangkok, Thailand; 2 Siriraj Center of Research Excellence for Diabetes and Obesity, Faculty of Medicine Siriraj Hospital, Mahidol University, Bangkok, Thailand; 3 Siriraj Center of Research Excellence Management, Faculty of Medicine Siriraj Hospital, Mahidol University, Bangkok, Thailand; 4 Research Division, Faculty of Medicine Siriraj Hospital, Mahidol University, Bangkok, Thailand; 5 Department of Immunology, Faculty of Medicine Siriraj Hospital, Mahidol University, Bangkok, Thailand

**Correction: Diabetologia**

10.1007/s00125-025-06373-y

The word ‘individuals’ was incorrectly repeated in the title. The original article has been corrected.

