# Supplementary material for: Islet autoantibodies in Thai individuals diagnosed with type 1 diabetes before 30 years of age: a large multicentre nationwide study
Source: Diabetologia. 2025 Feb 19;68(5):961–8. doi: 10.1007/s00125-025-06373-y (PMC12021985; doi:10.1007/s00125-025-06373-y)

ESM Table 1: Number of childhood (0-17 years) and young adulthood (18-30 years) patients

	0-17 years (n)	18-30 years (n)
Type 1 diabetes	604	192
Autoantibody positive type 1 diabetes	430	94
Autoantibody negative type 1 diabetes	174	98

ESM Table 2: The difference between the observed and expected autoantibody frequencies across the regions were analyzed using the Chi-square test.

Region	All 3	GAD65/ IA2	GAD65	GAD65/ ZnT8	IA2	IA2/ZnT8	ZnT8
C	9.62	6.28	-16.88	-1.82	3.10	0.63	-0.92
N	-0.15	-0.57	0.78	-1.05	1.35	-0.09	-0.27
NE	-0.23	-2.95	3.44	5.03	-3.65	-1.98	0.35
S	-9.23	-2.75	12.66	-2.17	-0.80	1.45	0.85

C, Centra; N, Northern; NE, Northeast; and S, Southern

ESM Fig. 1: Flow chart of recruitment in this study. T1DDAR CN = Thai Type 1 Diabetes and Diabetes Diagnosed Before 30 Years Registry, Care and Network, AAb- = autoantibody negative, AAb+ = autoantibody positive

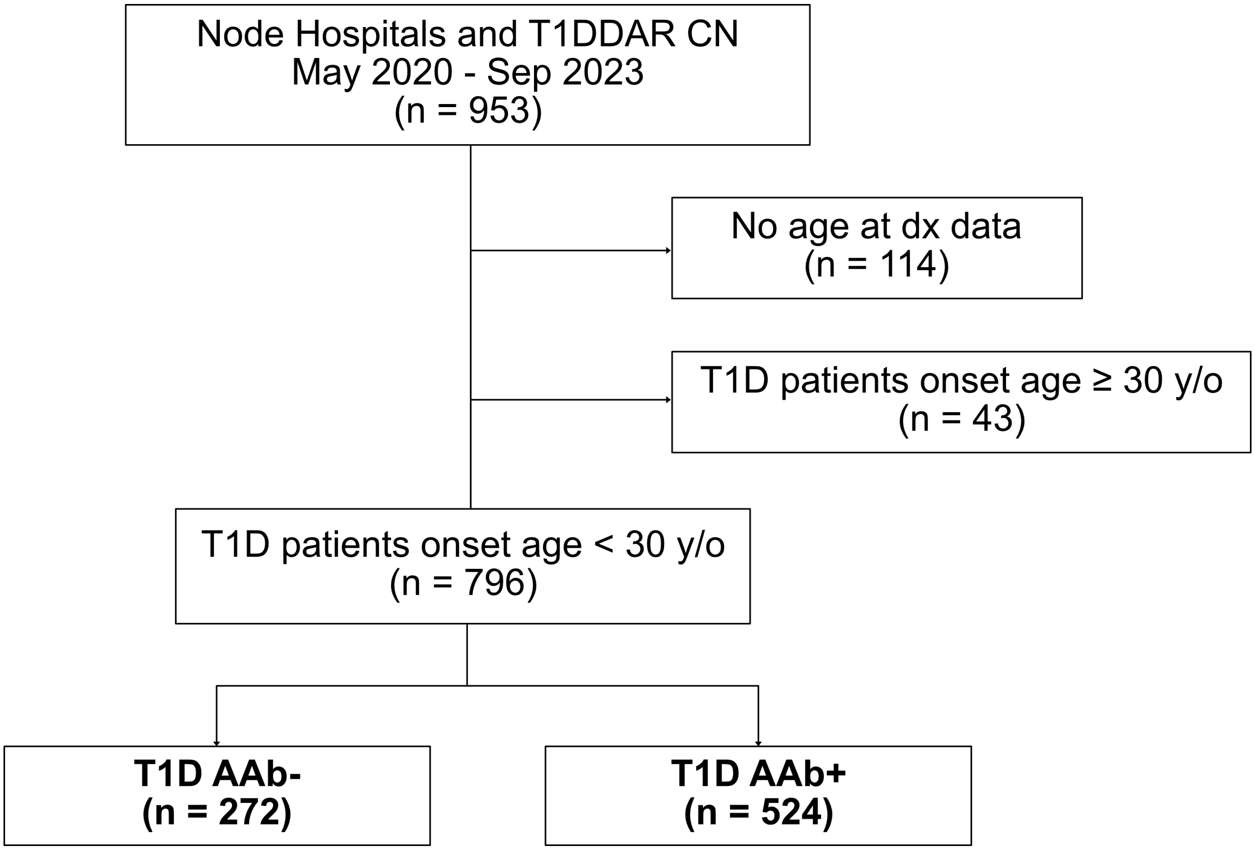

ESM Fig. 2: The Kernel Density Estimator (KDE) of autoantibodies in the Thai population and association between sex and autoantibody levels.

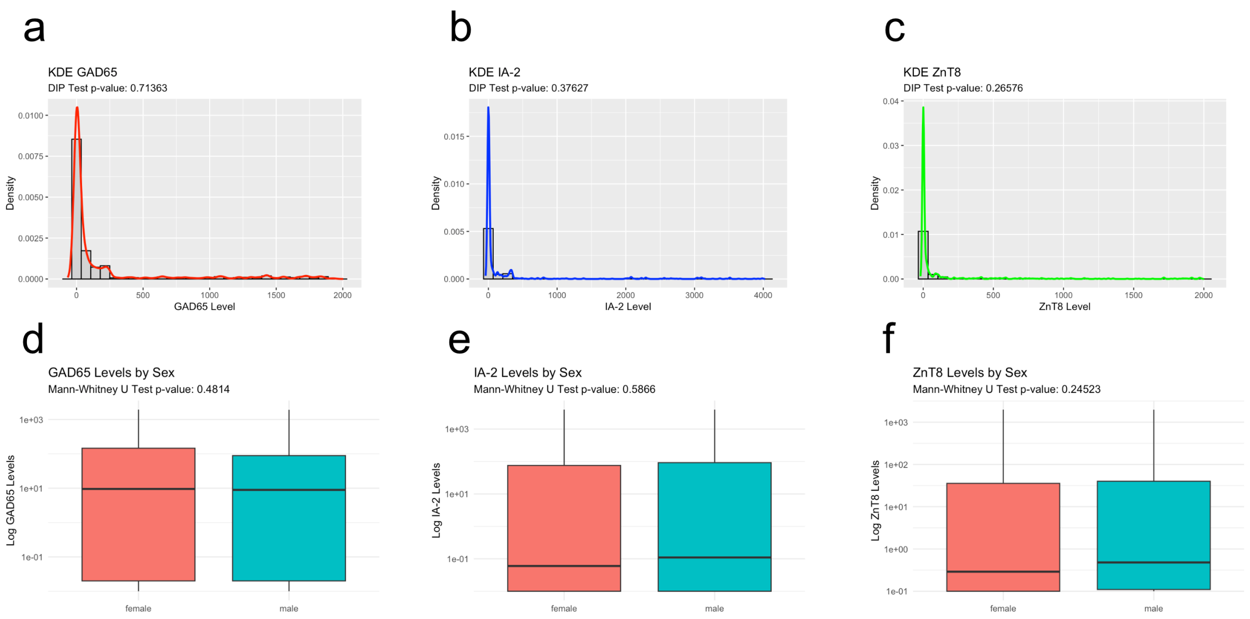

ESM Fig. 3: Count of random C-peptide levels < 0.6 ng/mL in each type 1 diabetes subgroup.

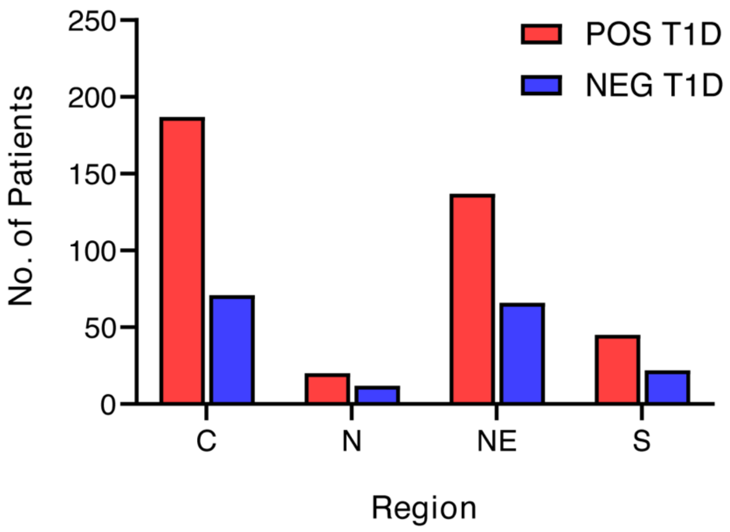

Supplement: Supplementary file 1 — ESM (PDF 743 KB) [file 125_2025_6373_MOESM1_ESM.pdf]
